# Capacitors Based on Polypyrrole Nanowire Electrodeposits

**DOI:** 10.3390/polym14245476

**Published:** 2022-12-14

**Authors:** A. M. R. Ramírez, M. A. del Valle, E. Ortega, F. R. Díaz, M. A. Gacitúa

**Affiliations:** 1 Centro de Nanotecnología Aplicada, Facultad de Ciencias, Ingeniería y Tecnología, Universidad Mayor, Camino la Pirámide 5750, Santiago 8580745, Chile; 2 Núcleo de Química y Bioquímica, Facultad de Ciencias, Ingeniería y Tecnología, Universidad Mayor, Camino la Pirámide 5750, Santiago 8580745, Chile; 3 Laboratorio de Electroquímica de Polímeros, Pontificia Universidad Católica de Chile, Av. V. Mackenna 4860, Santiago 7820436, Chile; 4 R&D Department, Leitat Chile, Román Díaz 532, Santiago 7500724, Chile; 5 Centro de Excelencia en Nanotecnología (CEN) Chile, Román Diaz 532, Santiago 7500724, Chile; 6 Facultad de Ingeniería & Ciencias, Universidad Diego Portales, Ejercito 441, Santiago 8370191, Chile

**Keywords:** capacitors, polymer nanowires, polymer electrosynthesis, polymer nanowire electrosynthesis, polypyrrole, polypyrrole nanowires

## Abstract

The electrochemical polymerization of polypyrrole nanowires is carried out using potentiodynamic and galvanostatic methods in order to enhance the performance of the modified electrodes as capacitor devices. The electrochemical, spectroscopic, and morphological properties are determined through cyclic voltammetry, Raman spectroscopy and scanning electron microscopy, respectively, corroborating the presence of PPy-nw in dimensions of 30 nm in diameter. Characterization as a capacitor revealed that the nanowire structure enhances key parameters such as specific capacitance with 60 times greater value than bulk polymer modification, in addition to a significant increase in stability. In this way, it is verified that electrodes modified with polypyrrole nanowires obtained in situ by electrochemical methods constitute an excellent candidate for the development of capacitors.

## 1. Introduction

In recent decades, conducting polymers (CP) have occupied an important place in the study of new types of materials, due to their different properties, which has led to the development of a wide range of electronic devices, such as electrochemical sensors [1,2], OLED (organic light emitting diodes) [3], photovoltaic cells [4,5], rechargeable batteries [6,7,8] and capacitors [9,10]. In the case of these last devices, conducting polymers stand out in comparison with inorganic compounds due to their high specific capacity, which can reach up to 2000 F g^−1^ in the case of polyaniline in sulfuric acid [11]. The most studied CPs are Polypyrrole (PPy), Polyaniline (PAni), Polythiophene (PTh), and their derivatives such as Poly(3,4-ethylenedioxythiophene) (PEDOT) because they present high conductivity in the doped state, with values of up to 500 S cm^−1^ [12,13]. However, polypyrrole (PPy) appears as one of the most promising polymers since its chemical properties make it possible to rapidly oxidize its monomer [14], both chemically [15] and electrochemically [16]. The latter is the most suitable because it can be performed in a controlled manner due to the well-described electrochemical polymerization mechanism [17,18], which directly relates the electrochemical perturbation type and the properties obtained, allowing the use of different doping anions [19], without losing their redox properties.

In this way, it has been reported that the electrodes constituted by CPs display better capacitance and processability, and greater cyclability—attributed to the improvement of the mechanical stability provided by the carbon-based compounds—used in the synthesis [20]. For instance, Gan et al. [21] reported high specific capacity and faster charging/discharging of prepared polypyrrole/graphene-coated carbon nanofiber electrode (CNF/PPy/GC). It is important to note that the high capacity is due to the presence of nanostructures of PPy—obtained inside the structures of the GC—indicating that the nanostructured CP has a high diffusion of the ions into the polymer. However, the increase in volume that PPy offers when generating the *p*-doping process typically damages the three-dimensional structure of graphene, producing an internal resistance, which ultimately damages the electrode [21]. Therefore, the role of CP nanostructures in supercapacitors is to decrease the dead volume when using bulk CPs electrodes. In this manner, in recent decades, the introduction of nanoscience and its application in various types of materials has generated an important advance in the development of new technologies, the main reason being the increase in surface area without losing properties. Compared to CP in its bulk form, nanostructures have even presented an increase of up to 300 times more in their *p*-doping/undoping charge [22].

In the present report, a method is proposed and tested to electrosynthesis PPy nanowires in situ from the electro-obtaining of mesoporous silicon oxide films reported by Walcarius et al. [23,24,25]. First, a thin layer of polymer is first electrodeposited on the electrode, followed by electrochemical polycondensation of a mesoporous silicon oxide, which will act as a template for the subsequent electrosynthesis of the polymer nanostructures. Thus, once the template is removed, the thin polymer layer provides a stable covalent bonded support, ensuring high stability and repeatability. Another important advantage of this method is that is carried out directly in situ [22,26,27,28].

Based on this background, the PPy *p*-doping/undoping process is compared between nanostructured and bulk morphology, obtaining on electronic properties, with application in energy storage devices, such as capacitors.

## 2. Materials and Methods

The modification of ITO to prepare the ITO|PPy and ITO|PPy, PPy-nw electrodes is performed using past reported methods [22]. Electrochemical polymerization is conducted in three-compartmentanchor-type glass cells (Own confection, Santiago, Chile), using as a working electrode a conductive glass coated with indium-doped tin oxide, ITO (PGO 10 Ω cm^−1^) cleaned and degreased in ultrasound for 5 min in acetone, 5 min in propanol, and finally, 5 min in milli-Q water. A large area spiral Pt wire is used as a counter electrode, and an Ag|AgCl electrode with potential adjusted to that of SCE, manufactured in-house, is used as a reference [29]. Polymerization is made in a solution containing 0.010 mol L^−1^ of PPy with 0.100 mol L^−1^ Tetrabuthylammonium hexafluorophosphate, TBAPF_6_, in anhydrous CH_3_CN. Electrochemical characterization of the *p*-doping/undoping process was carried out through 1000 successive voltammetric cycles (*n* = 1000) at a potential scan rate, ν, of 0.1 V s^−1^. The characterization as capacitor devices is made through analyzes of specific capacitance, retention capacity, and stability of the charge/discharge process in an aqueous system consisting of 0.1 mol L^−1^ of LiCl for 1000 successive cycles. All measurements were carried out under a high-purity argon atmosphere and at room temperature, 20 °C.

The value of the specific capacitance (*C*) for the bulk and nanostructured polypyrrole-modified electrode is calculated from cyclic voltammetry using Equation (1) [30]:

(1) $$C=\frac{\int_{E_{1}}^{E_{2}}i\;\left(E\right)d\left(E\right)}{2vm\left(E_{2}-E_{1}\right)}\;$$

where *E_2_* and *E_1_* are the inversion potentials of the cyclic voltammetry, The value of $\int_{E_{1}}^{E_{2}}i\;\left(E\right)d\left(E\right)\;$ corresponds to the total charge (positive and negative sweep), *V* is the scan rate, and *m* is the mass of the polymer, which was calculated from the methodology described by del Valle et al. [19,31]. On the other hand, the specific capacitance calculated from the charge–discharge method is carried out with Equation (2) [32]:

(2) $$C=\frac{it}{mV}\;\;$$

where *C* is the specific capacitance (F g^−1^), it is the product of the discharge current and time, respectively, *V* is the potential range of the measurement, and *m* is the mass of the polymer. All electrochemical measurements were made using a potentiostat/galvanostat CH Instruments 900B (CH Instruments, Inc., Austin, TX, USA).

The Raman spectroscopy of CP was measured in a range of 100 to 2000 cm^−1^ in a MultiRam spectrometer (Bruker Optics GmbH & Co. KG., Ettlingen, Germany) with Nd:YAG laser as an excitation source (λexc = 514.5 nm), controlled by Opus software (Opus Software, North York, TO, Canada). All experiments were conducted at room temperature, in the air on thin films freshly deposited.

The morphology of the modified electrode was observed by scanning electron microscopy, SEM, initially depositing 5 nm of gold using sputtering and applying 10 KeV through an Inspect F50, FEI microscope (FEI Company, Eindhoven, The Netherlands).

## 3. Results and Discussion

**Figure 1:**
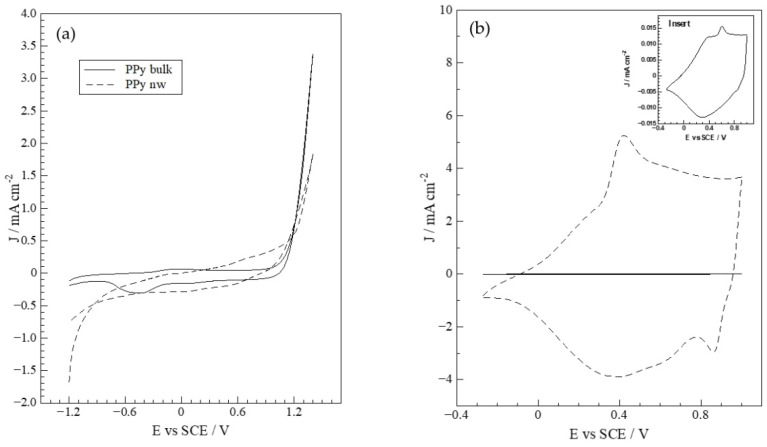
(**a**) Cyclic voltammograms during growth of PPy on modified ITO electrodes|polymer in: (––) absence of template, and (- - -) template presence. (**b**) *p*-doping/undoping study in 0.100 mol L^−1^ TBAPF_6_, CH_3_CN of ITO modified with: (––) bulk PPy, (- - -) PPy-nw. ν = 0.100 V s^−1^. Insert: zoom of PPy bulk *p*-doping/undoping study.

Voltammetric profiles corresponding to the electrosynthesis of PPy in the absence and presence of a template are shown in Figure 1a, producing ITO electrodes modified with conventional PPy, PPy bulk, and nanostructured PPy, PPy-nw, respectively [22]. In Figure 1b, the shape of the *p*-doping/undoping profile presents a rectangular wave, indicating ideal capacitive behavior [33,34,35]. Then, the intensity of this *p*-doping/undoping process of PPy bulk and PPy-nw is compared. PPy bulk presents a *p*-doping/undoping current density negligible compared to that of PPy-nw. This behavior is consistent with the significant increase in effective area attributable to the nanostructuring of the deposit on the same support area, similar to outcomes from different polymers in past reports [4,22,26,36,37,38,39,40,41].

The *p*-doping/undoping CV profile in anhydrous conditions, Figure 1b, presents considerable difference in current density and position for doping processes. The enhancement of surface area may explain the increase in current density due to nanowire morphology. Additionally, the potential shift implies that nanostructuring somehow decreases the energy required for the doping process to take place, i.e., the arrangement of the nanowires thermodynamically favors the *p*-doping process, as has been described in previous reports for analogous systems [28,40,42].

The effect of electrode structure on the description of doping process is presented on Table 1. It can be verified that ITO|PPy-nw produces 300 times the charge of ITO|PPy, a predictable outcome considering the nanostructured surface of the wires. In addition, it is important to note the doping process is completely reversible since, for both ITO|PPy and PPy-nw the ratio between the *p*-doping and p-undoping charges is practically 1, implying that the nanometric morphology does not affect stability and/or durability of the conducting polymer film [43].

Moreover, the Raman characterization of the deposits (Figure S1, Table S1) shows the presence of characteristics polaron and bi-polaron formation bands at 972 and 935 cm^−1^, res since according to literature, these bands have been assigned to polarons and bi-polarons formation [44]. In other words, its presence would mean partial oxidation of the polymer chain. On the other hand, it is important to note that the bands described in Table S1 are intrinsic to the PPy structure. Additionally, the ratio between the area of the polarons and bipolarons signals, as well as C=C and the sum of polaron and bipolarons, is always higher for PPy-nw modified electrodes (Table S2), as described in the literature for conductive polymers [45].

**Figure 2:**
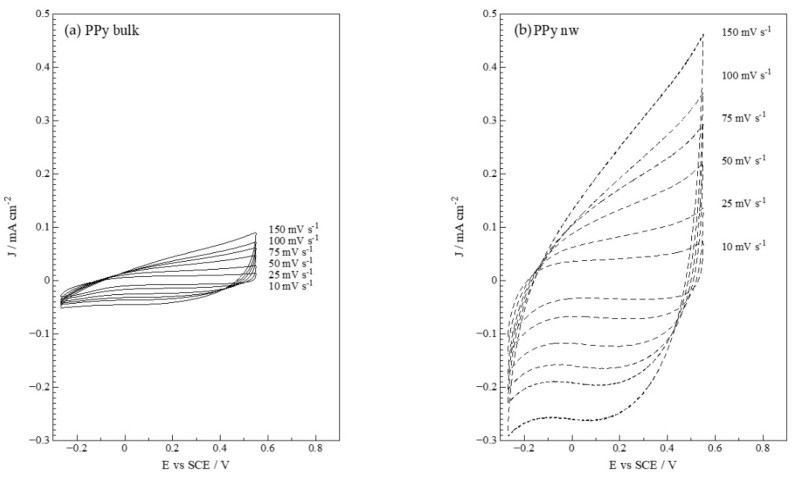

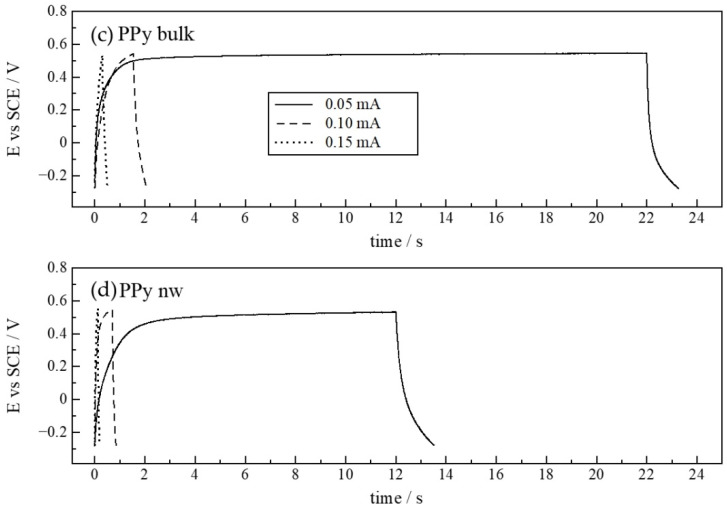
Electrode characterization in aqueous medium with LiCl 0.100 mol L^−1^: (**a**,**b**) cyclic voltammetry at different scan rates. (**c**,**d**) Galvanostatic measurements at different currents.

Then, to check and compare the applicability of ITO|PPy bulk and ITO|PPy-nw as capacitors, their electrochemical response is analyzed in aqueous 0.1 mol L^−1^ LiCl, Figure 2a,b.

The CV response for the *p*-doping/undoping processes in an aqueous LiCl-containing medium at different scan rates displays good stability and the desired rectangle shape. Current density attained by the nanowired electrodes is much higher than that from the bulk deposit. Additionally, relevant research has reported that when including nanostructures such as graphene oxide into electrode architecture, the current values attained were remarkably higher than regular PPy deposit [33,34]. The authors indicate that the incorporation of nanostructures enhances electrode porosity and increases electroactive area, explaining the current increment. However, with respect to the scan-rate study, Yang et al. [33] report show that faster rates modify the voltammogram shape and peak position due to longer ion diffusion time with respect to polarization. This is not the case for the present study; CV profiles in LiCl present a stable profile without significant peak position shifts at higher scan rates. This indicates that while including nanostructures such as graphene oxide certainly increases electrode active surface, it does not necessarily improve charge/discharge reversibility as nanowires do.

Galvanostatic measurements in an aqueous LiCl-containing medium, Figure 2c,d show asymmetrical charge/discharge curves, including a potential plateau, characteristic for battery-type material [46]. At different current perturbation values, the charge and discharge processes between −0.27 and 0.50 V vs. SCE is presented. It is clear that at higher currents, the charge and discharge time in the measured potential range decreases and the charge process takes place much faster in the nanowired deposit, more noticeable at low currents.

On the other hand, when comparing the galvanostatic charge and discharge measurements of the modified electrodes (Figure S2 in Supplementary Materials), it is observed that the presence of PPy-nw generates a faster charge/discharge response than PPy bulk in the intervals of measured potential. This behavior can be explained mainly by the ease of PPy-nw for the *p*-doping process, as already deduced from the data in Table 1. The high order degree of the nanostructured film increases electrode process speed, as has been reported for different deposits with similar morphology and methods [27,28,40,42].

From the potentiodynamic voltammograms and the galvanostatic potential/time transients at Figure 2, the specific capacitance of the modified electrodes is calculated, see Table 2.

For CV measurements, at lower scan rates, the calculated specific capacitance increases, and the electrode modified with PPy-nw presents a value 100 times greater than the respective electrode with PPy bulk. This is explained by the significant increase in active area of the nanostructured electrode on the same geometric area of the ITO support electrode. Additionally, the value of the charge decreases as the speed of the scan increases, which is related to the diffusion and migration of ions. However, the percentages of the specific capacitance with respect to that at 10 mVs^−1^ for the PPy-Bulk and PPy-nw modified electrodes are 68.6%, 52.4%, 41.9%, 34.3%, 27.6% and 73.8%, 58.2%, 49.6%, 42.2%, and 36.3%, respectively, for 20, 50, 75, 100, and 150 mVs^−1^, indicating the better diffusion of ions in the nanostructure due to the decrease in the dead volume [47]. From the galvanostatic measurements, it is verified, as from voltametric measurements, that the electrodes modified with PPy-nw present almost 60 times greater capacitance than those modified with PPy bulk. In addition, this value would be affected by the magnitude of the current applied to the system, verifying that the higher the current, the lower the specific capacity, which would be related to the charge and discharge time generated. This is also checked by Yang et al. [33] when using graphene oxide nanosheets to modify electrodes with PPy for capacitor design.

Moreover, galvanostatic measurements during 1000 cycles using PPy bulk and nw electrodes were also considered (Figure S3, Supplementary Materials). In both cases, a shift in the potential range towards more positive values is evident as a function of the number of cycles. However, for PPy bulk-modified electrodes, the amplitude of the potential range is 190 mV greater than the initial one, while for PPy-nw-modified electrodes, this amplitude only increases 30 mV with respect to the initial value. This increase could respond to the decrease in the conductivity of the polymer due to its over-oxidation.

**Figure 3:**
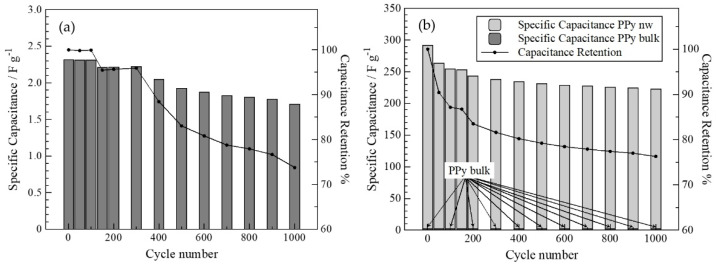
Graphic representation of specific capacitance and % of capacitance retention vs. number of charge/discharge cycles obtained in galvanostatic measurements in 0.1 mol·L^−1^ LiCl for 1000 cycles: (**a**) PPy bulk, and (**b**) PPy-nw.

If the specific capacitance is calculated after each galvanostatic charge cycle and presented with the capacitance retention (the percent of retained initial specific capacitance) versus the cycle number, Figure 3 shows the stability through the charge/discharge process can be evaluated. In the figure, PPy-nw presents a much bigger specific capacitance than its bulk homologue. Similarly, an enhanced specific capacitance has been presented in the literature when PPY electrodes were combined with graphene oxide nanosheets [33]. Therefore, the increment of specific capacitance when considering nanostructures on electrode architecture is observed on this work and on those from other authors [33,34]. Most likely, the larger active area offered by nanostructures (nanowires, graphene oxide) produces significant increase in parameters such as calculated specific capacitance.

On the other hand, the evolution of capacitance retention is similar in both cases, indicating that nanowire morphology does not affect the stability of these device, but rather, it would only generate a significant increase in capacity. In parallel, this behavior is also observed in the charge and discharge measurements performed by cyclic voltammetry for 1000 cycles using an identical solution (Supplementary Materials Figure S4).

**Figure 4:**
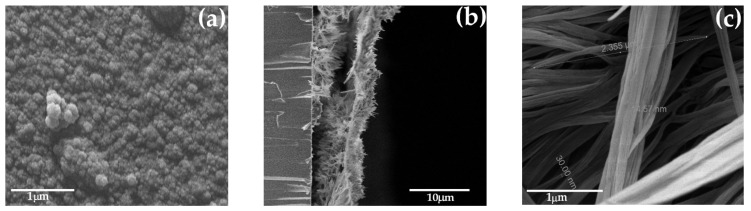
SEM micrographs of PPy: **(a)** bulk, and **(b)** and **(c)** nanowires.

Then, to corroborate the nanostructured morphology, scanning electron micrographs were obtained and analyzed, Figure 4. The frontal view of PPy bulk presents a granular growth, characteristic of PPy prepared under these conditions [19]. On the other hand, the PPy-nw side and front views, Figure 4b,c display the formation of 15–30 nm wide nanowires. Additionally, to its morphology, the deposits show excellent adherence, as previously verified [26,28], increasing easy manipulation and durability, useful characteristics in energy storage devices.

Finally, to evaluate the potentiality of the application of PPy-nw in the construction of supercapacitor devices Table 3 shows a summary of common parameters for capacitor characterization from other authors [33,34,35] with similar materials compared to those obtained in present work.

The performance of the capacitors reported in the present work has comparable indicators with others from different authors. For example, the specific capacitance is at least 1500 times higher than that found for the graphene oxide PPy composite on glassy carbon electrode as from Yang et al. [33]. On the other hand, the performance obtained by the device from Zhang et al. [33] consisting of a composite between graphene oxide and chemically polymerized PPs is quite similar to this work. Nevertheless, the incorporation of graphene oxide on electrode architecture irremediably increases the device cost efficiency. Additionally, reports from other authors [33,34,48] present higher capacitance retention than present work. The reason for this, as stated by Zhang and Zhao [34], could be that the combination of rigid structures (GO and CDC) on electrodes enhances the mechanical strength of composites, preventing swelling and shrinking during long-term cycling. This is not the case in present work, since PPy-nw are not combined with other rigid components.

On the other hand, capacitor stability is comparable to reports from other authors with similar materials. This behavior is attributed to the fact that the electrodes reported in the literature are additionally modified with GO, which aims to improve stability [51].

In general terms, the outcomes from this study present a simple way to produce nanostructured supercapacitors based on electrosynthesized PPy nanowires with overall better performance when compared to similar devices from different authors. The methods are repeatable and may be applied to different polymeric systems (polythiophene, polyaniline or poly3,4-ethylenedioxythiophene), to compare and eventually optimize these results.

## 4. Conclusions

ITO electrodes are modified in situ with polypyrrole nanowires by means of electrochemical steps. Cyclic voltammetry was used characterize the difference between bulk and nanostructured PPy by comparison of voltammograms during synthesis and doping processes; PPy-nw displayed higher currents and lower peak potential values if compared to bulk deposits, which translated on higher specific capacitance as well. Galvanostatic measurements in aqueous LiCl served to characterize the potential of the deposit in the design of capacitors. Nanostructured PPy showed 60 times higher specific capacitance, superior stability through 1000 charge/discharge cycles, and reproducibility compared to its bulk form. After analyzing SEM micrographs of the deposit, it was observed that PPy-nw deposit contain 15–30 nm width wire in a brush-like disposition normal to ITO electrode surface.

The performance of the reported device is comparable to those found in literature from different authors with analogous systems. However, reports from other authors typically use graphene-based modifications, greatly enhancing the economic cost of the device in case of scalation to industrial prototypes. Consequently, the present outcomes were submitted as a patent application.

## 5. Patents

The authors applied for an international patent called “Electrosynthesis of polymeric nanowires directly on solid surfaces (electrodes)”, with application numbers PCT/CL2018/050116 and reference 273176-WO.

## Figures and Tables

**Table 1:** Effect of electrode structure on *p*-doping process description.

Electrode	E_p_ ^1^/V	Q_d_ ^2^ (mC cm^−2^)	Q_ud_ ^3^ (mC cm^−2^)	Q^d^/Q_ud_ ^4^
PPy bulk	0.60	0.10	0.10	1.04
PPy-nw	0.42	31.8	30.5	1.04

^1^ Potential; ^2^ *p*-doping charge; ^3^ undoping charge; ^4^ doping/undoping charge ratio.

**Table 2:** Effect of electrode structure on calculated specific capacitance.

		**Potentiodynamic**						
**Scan Rates (mV s^−1^)**			**10**	**20**	**50**	**75**	**100**	**150**
PPy bulk		Specific Capacitance (F g^−1^)	1.05	0.72	0.55	0.44	0.36	0.29
PPy-nw			102.6	75.7	59.7	50.9	43.3	37.2
		**Galvanostatic**						
**Current (mA)**			**0.05**		**0.10**		**0.15**	
PPy bulk		Specific Capacitance (F g^−1^)	4.86		3.84		3.49	
PPy-nw			263.9		217.4		200.9	

**Table 3:** Comparison of capacitive characteristics for other reports with similar materials.

Electrode Architecture	Perturbation Type and Intensity	Electrolyte	Specific Capacitance	Capacitance Retention%, Charge Cycle Number and Charge Potential	Ref.
GC|GO/PPy ^1^	G ^3^: 1 mA cm^−2^	KCl	196 mF cm^−1^	90% for 500 cycles and 1.0 V	[33]
Au|GO/PPy ^2^	G ^3^: 0.3 A g^−1^	H_2_SO_4_	249 F g^−1^	81% for 1000 cycles and 1.0 V	[34]
FTO|PPy ^1^	P ^4^: 20 mV s^−1^	SDBS	12 F g^−1^	-	[35]
FTO|PPy ^1^	G ^3^ 2 mA cm^−2^	SDBS	7 F g^−1^	-	[35]
PPyCDC-EG ^1^	G ^3^ 2 A g^−1^	NaClO_4_	190 F g^−1^	90% for 1000 cycles and 0.3 V	[48]
PPy(SDBS)CDC ^1^	P ^4^: 10 mV s^−1^	SDBS	131 F g^−1^	-	[49]
PPy-PEO/DBS ^1^	G ^3^ 0.24 Ag^−1^	NaCl	100 F g^−1^	-	[50]
ITO|nw-PPy ^1^	G ^3^: 0.15 mA	LiCl	291 F g^−1^	76.5% for 1000 cycles and 0.77 V	This work

^1^ Electrochemical polymerization, ^2^ chemical polymerization, ^3^ galvanostatic, ^4^ potentiodynamic, SDBS = sodium dodecylsulfate, CDC = Carbide derived carbon, PEO = Poly (ethylene oxide), EG = ethylene glycol.

## Data Availability

Not applicable.

## Supplementary Materials

The following supporting information can be downloaded at: https://www.mdpi.com/article/10.3390/polym14245476/s1, Figure S1: Raman spectra of bulk and nanowire PPy ITO modified electrodes; Table S1: Raman spectrum assignments of bulk and nanowire PPy; Table S2: Areas calculated from Raman spectroscopy signals; Figure S2: Galvanostatic measurements of charge and discharge of bulk and nanowire PPy deposits in 0.100 mol L^−1^ LiCl at 0.15 mA; Figure S3: Galvanostatic measurements of charge and discharge in 0.100 mol L^−1^ LiCl: (a,b) PPy bulk, and (c,d) PPy-nw for i = 0.15 mA; Figure S4: Graphic representation of specific capacitance and % of capacitance retention vs. number of charge/discharge cycles obtained by cyclic voltammetry in 0.1 mol L^−1^ aqueous LiCl solution for 1000 successive cycles: (a) ITO|PPy, and (b) ITO|PPy,PPy-nw.
