# Supplementary material for: Capacitors Based on Polypyrrole Nanowire Electrodeposits
Source: Polymers (Basel). 2022 Dec 14;14(24):5476. doi: 10.3390/polym14245476 (PMC9782085; doi:10.3390/polym14245476)
Supplement: Supplementary file 1 [file polymers-14-05476-s001.zip › polymers-2083231-supplementary.pdf]

SUPPLEMENTARY INFORMATION

Capacitors Based on Polypyrrole Nanowire Electrodeposits

A. M. R. Ramírez [1,2,*], M. A. del Valle [3], E. Ortega [4,5], F. R. Díaz [3] and M. A. Gacitúa [6,*]

1 Centro de Nanotecnología Aplicada, Facultad de Ciencias, Ingeniería y Tecnología, Universidad Mayor, Camino la Pirámide 5750, Santiago 8580745, Chile
2 Núcleo de Química y Bioquímica, Facultad de Ciencias, Ingeniería y Tecnología, Universidad Mayor, Camino la Pirámide 5750, Santiago 8580745, Chile
3 Laboratorio de Electroquímica de Polímeros, Pontificia Universidad Católica de Chile, Av. V. Mackenna 4860, Santiago 7820436, Chile
4 R&D Department, Leitat Chile, Román Díaz 532, Santiago 7500724, Chile
5 Centro de Excelencia en Nanotecnología (CEN) Chile, Román Diaz 532, Santiago 7500724, Chile
6 Facultad de Ingeniería & Ciencias, Universidad Diego Portales, Ejercito 441, Santiago 8370191, Chile
* Correspondence: andres.ramirez@umayor.cl (A.M.R.R.); manuel.gacitua@udp.cl (M.A.G.)

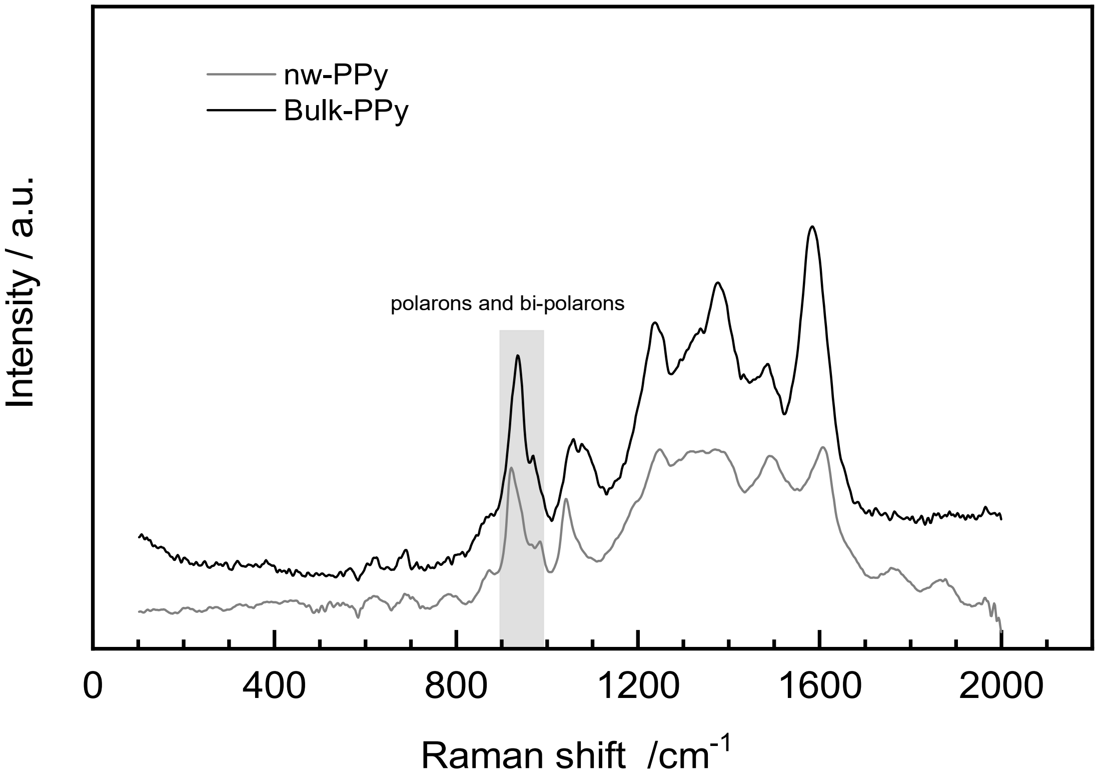

Figure S1. Raman spectra of bulk and nanowire PPy ITO modified electrodes.

Table S1. Raman spectrum assignments of bulk and nanowire PPy.

ITO|PPy		ITO|PPy,PPy-nw
**wavelenght (cm^{-1})**	**assignment**	**wavelenght (cm^{-1})**
1075	C–H characteristic	1042
1246	C_{α}–N stretching	1244
1337	C_{α}–C_{α} (inter rings between chains) stretching	1314
1379	C_{β}–C_{β} ring stretching	1380
1486	symmetrical C_{α}=C_{β} stretching	1490
1583	asymmetric C_{α}=C_{β} stretching	1600

Table S2: Areas calculated from Raman spectroscopy signals.

	935 cm^{-1}	972cm^{-1}	ratio 972/935	1600 cm^{-1}	ratio 1600/(935 +972)
PPy-Bulk	12.5303	9.8064	0.783	28.0493	1.256
PPy-Nw	6.2162	5.5577	0.895	15.067	1.280

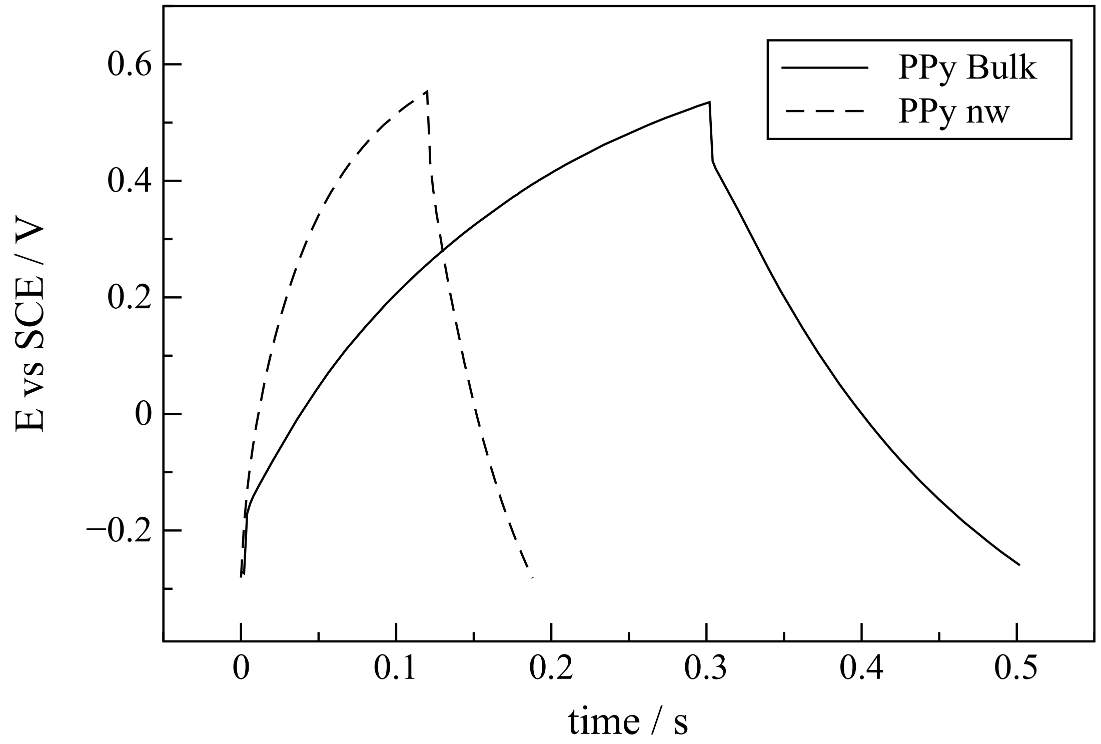

Figure S2. Galvanostatic measurements of charge and discharge of bulk and nanowired PPy deposits in 0.100 mol L^{-1} LiCl at 0.15 mA.

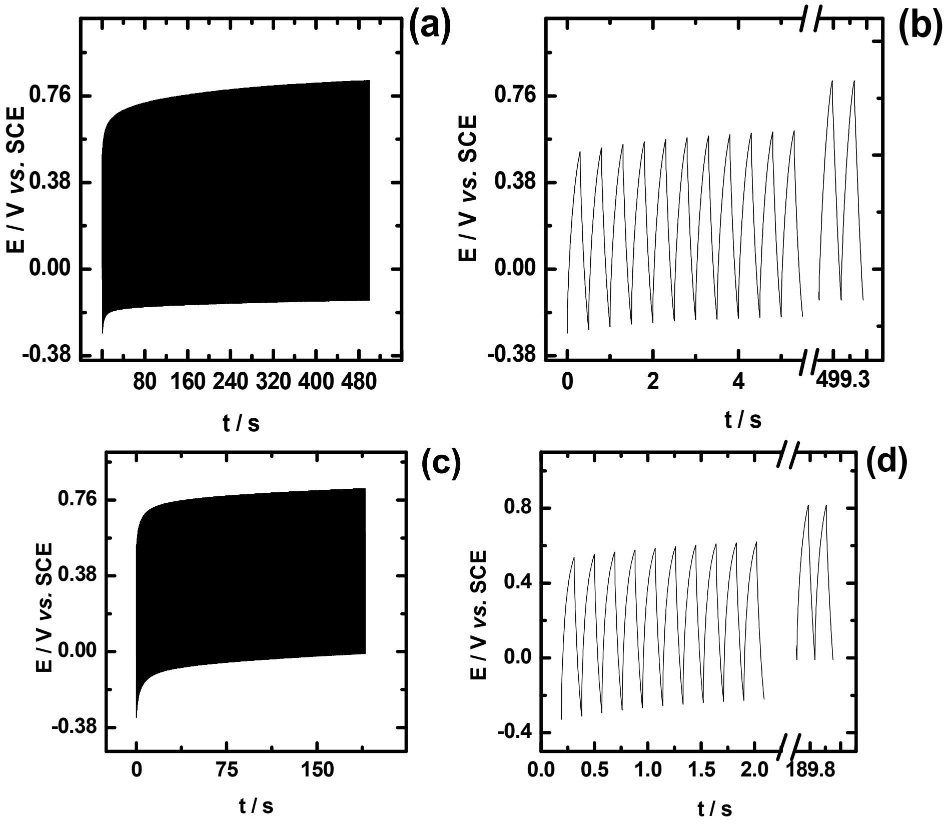

Figure S3. Galvanostatic measurements of charge and discharge in 0.100 mol L^{-1} LiCl: (a,b) PPy bulk, and (c,d) PPy-nw for i = 0.15 mA.

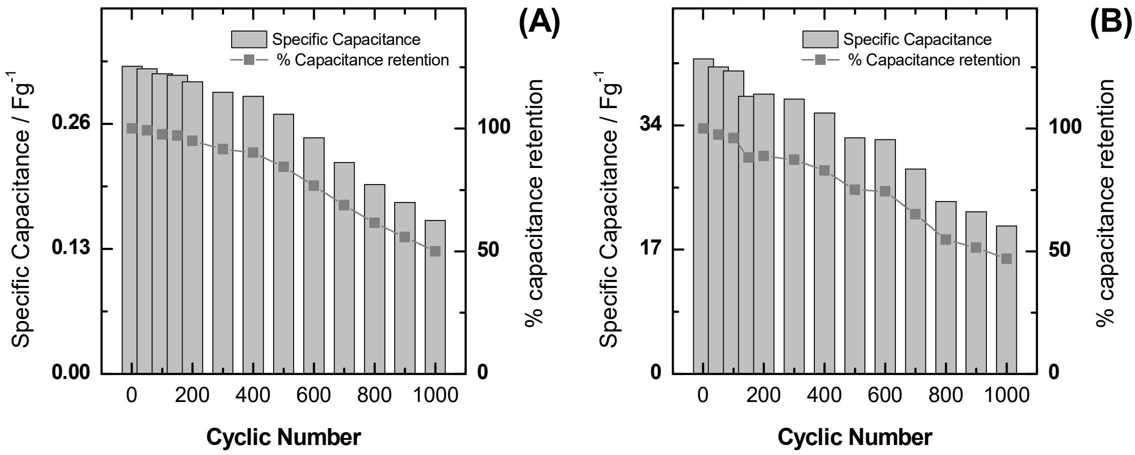

Figure S4. Graphic representation of specific capacitance and % of capacitance retention *vs*. number of charge/discharge cycles obtained by cyclic voltammetry in 0.1 mol L^{-1} aqueous LiCl solution for 1000 successive cycles: (a) ITO|PPy, and (b) ITO|PPy,PPy-nw.
